# Particles act as ‘specialty centers’ with expanded enzymatic function throughout the water column in the western North Atlantic

**DOI:** 10.3389/fmicb.2022.882333

**Published:** 2022-09-28

**Authors:** C. Chad Lloyd, Sarah Brown, John Paul Balmonte, Adrienne Hoarfrost, Sherif Ghobrial, Carol Arnosti

**Affiliations:** ^1^Department of Marine Sciences, University of North Carolina at Chapel Hill, Chapel Hill, NC, United States; ^2^Environment, Ecology and Energy Program, University of North Carolina at Chapel Hill, Chapel Hill, NC, United States; ^3^Department of Biology, HADAL and Nordcee, University of Southern Denmark, Odense, Denmark; ^4^Department of Marine Sciences, University of Georgia, Athens, GA, United States

**Keywords:** organic matter, polysaccharide hydrolase, peptidase, biological pump, community composition, mesopelagic, bathypelagic

## Abstract

Heterotrophic bacteria initiate the degradation of high molecular weight organic matter by producing an array of extracellular enzymes to hydrolyze complex organic matter into sizes that can be taken up into the cell. These bacterial communities differ spatially and temporally in composition, and potentially also in their enzymatic complements. Previous research has shown that particle-associated bacteria can be considerably more active than bacteria in the surrounding bulk water, but most prior studies of particle-associated bacteria have been focused on the upper ocean - there are few measurements of enzymatic activities of particle-associated bacteria in the mesopelagic and bathypelagic ocean, although the bacterial communities in the deep are dependent upon degradation of particulate organic matter to fuel their metabolism. We used a broad suite of substrates to compare the glucosidase, peptidase, and polysaccharide hydrolase activities of particle-associated and unfiltered seawater microbial communities in epipelagic, mesopelagic, and bathypelagic waters across 11 stations in the western North Atlantic. We concurrently determined bacterial community composition of unfiltered seawater and of samples collected *via* gravity filtration (>3 μm). Overall, particle-associated bacterial communities showed a broader spectrum of enzyme activities compared with unfiltered seawater communities. These differences in enzymatic activities were greater at offshore than at coastal locations, and increased with increasing depth in the ocean. The greater differences in enzymatic function measured on particles with depth coincided with increasing differences in particle-associated community composition, suggesting that particles act as ‘specialty centers’ that are essential for degradation of organic matter even at bathypelagic depths.

## Introduction

The formation and dynamics of particles in the ocean are an important facet of the marine carbon cycle. Particles sinking out of the surface ocean drive the biological carbon pump, acting to modulate atmospheric CO_2_ concentrations by removing organic carbon from the epipelagic zone, and redistributing carbon, nutrients, and energy to deeper depths (Herndl and Reinthaler, 2013; Turner, 2015). The fate of these particles—the extent to which they fuel heterotrophs in the mesopelagic and bathypelagic ocean, as well as the fraction of particles that reach the sediments—depends on the nature of the particles, the rates at which they sink, and the extent to which they are disrupted, transformed, re-aggregated, and respired in the water column (Passow and Carlson, 2012; Boyd et al., 2019). Given the importance of these processes, considerable effort has been focused on understanding particle dynamics, measurements of sinking flux, investigations of suspended/sinking transitions, and quantification of factors affecting rates of transformation and the extent to which particulate organic matter (POM) is remineralized with depth (De La Rocha and Passow, 2007; Abramson et al., 2010; Fender et al., 2019).

Since the fate of POM depends in part on the activities of the heterotrophic microbes that colonize the particles, the composition and role of particle-associated (PA) microbial communities have likewise been closely examined. Notable differences in the composition of PA and free-living microbial communities have been found in both the surface/mesopelagic (DeLong et al., 1993; Salazar et al., 2016; Valencia et al., 2022) and deep ocean (Eloe et al., 2011). Considerable effort has been devoted to characterizing the metabolic capabilities of microbial communities, typically *via* ‘omics methods that indicate the genetic potential for specific metabolic processes. These studies have revealed distinct depth stratification of organisms, genes, transcripts, and proteins (Mende et al., 2017; Bergauer et al., 2018); important differences in metabolic potential between PA and bulk communities have also been identified (Acinas et al., 2021). Understanding the role and function of microbial communities in carbon processing, however, also requires measurements of activities to identify the extent to which functional potentials are realized in the environment.

To determine rates of complex processes, a focus on key steps is required. The ability of heterotrophic microbes to access POM, for example, depends critically on the enzymatic repertoire of community members. HMW organic matter must initially be hydrolyzed by structure-selective extracellular enzymes to sizes sufficiently small to transport into the cell. This initial hydrolytic step is the gateway to further microbial processing of organic matter (reviewed in Arnosti, 2011). Experimental investigations have found high levels of enzymatic activities among PA microbial communities (Smith et al., 1992; Grossart et al., 2003a,b; Kellogg and Deming, 2014); particles have been identified as ‘hot spots’ of organic matter remineralization (Simon et al., 2002), which can lead to the production of dissolved organic matter that also fuels free-living organisms in close proximity to particles (Smith et al., 1992; Visser and Jackson, 2004).

Most studies investigating enzymatic function of PA microbial communities, however, have relied on a small set of substrate proxies—typically a monomer such as leucine or glucose linked to a fluorophore—to characterize enzyme activities (e.g., Grossart et al., 2003a; Kellogg and Deming, 2014). When the monomer-fluorophore bond is hydrolyzed, fluorescence increases, providing rapid information about the activity of exo-acting (terminal-unit cleaving) enzymes. These small substrate proxies, although providing data that can be inter-compared among studies, do not reflect the 3D structure of complex organic macromolecules, and do not capture the activities of endo-acting (mid-chain cleaving) enzymes essential to hydrolysis of complex organic matter. Thus, this approach misses significant functional aspects of microbial extracellular enzymes. Alternative approaches using fluorescently labelled polysaccharides measure the structural specificities and activities of both exo- and endo-acting enzymes, and have demonstrated notable differences by substrate, location, and depth in the ocean (Arnosti et al., 2011; Hoarfrost and Arnosti, 2017; Balmonte et al., 2021). These approaches have also been used in a handful of studies to investigate enzyme activities of PA communities (D’Ambrosio et al., 2014; Balmonte et al., 2018, 2020, 2021). However, few data are available on the enzymatic activities of PA communities in the bathypelagic ocean.

Given the importance of particles for microbial communities in the bathypelagic ocean (Bergauer et al., 2018), where they fuel approximately 90% of bacterial metabolism (Hansell et al., 2009), we investigated PA activities and communities at 11 stations in the western North Atlantic, at depths ranging from the epipelagic to the bathypelagic ocean. Investigating this depth gradient is particularly important since microbial communities and genetic potential differ with depth (DeLong et al., 2006; Mende et al., 2017; Bergauer et al., 2018), including the genetic potential to produce extracellular enzymes (Zhao et al., 2020). Since particle source, abundance, and nature vary considerably between nearshore and open ocean environments (Lam et al., 2015), we also compared spatial patterns and variability in enzyme function in a gradient of coastal and offshore stations. The composition and activities of communities collected *via* gravity filtration (thus including both suspended and sinking particles) were compared with those of the whole (unfractionated) water in epipelagic, mesopelagic, and bathypelagic waters, encompassing zones in which much of the surface-derived organic matter is reprocessed in the ocean. We assessed the ability of microbial communities to enzymatically access complex substrates—focusing on peptides and polysaccharides that are characteristic of some of the major constituents of POM (Kharbush et al., 2020)—and concurrently characterized the composition of the communities in the PA and bulk fractions from the same samples.

## Materials and methods

### Stations and water sampling

Water samples were collected during two cruises aboard the R/V *Endeavor* in the western North Atlantic. Samples were collected at one shelf break and two open ocean stations (Supplementary Table S1) between April 27 and May 2 2015 (cruise EN556), and at 9 stations generally along an E-W transect at 36° N, including three shelf- or shelf-break stations and six open ocean stations (EN584; June 29–July 11 2016, Supplementary Figure S1). In 2016, Stn. 12 was re-occupied 6 days after the first sampling and is noted as Stn. 12r. Water was sampled using a Niskin rosette equipped with a Seabird 911+ CTD, which monitored depth, temperature, salinity, chlorophyll-*a*, and oxygen (Supplementary Table S1). At Stns. 4, 5, and 8, surface and bottom water were collected; at Stns. 10–16, water was collected at the deep chlorophyll maximum, oxygen minimum zone (as determined *via* the oxygen sensor of the CTD, at depths ranging from *ca.* 645–900 m; Supplementary Table S2), and deep water at a depth of 1,500 m, except Stn. 10, where subsurface collection depths were 300 and 530 m, respectively, due to the location of the station; Supplementary Figure S2; Supplementary Table S2. Note that data from the subsurface depths of Stn. 10 was therefore plotted as mesopelagic. Stn. 9 water was collected only at the surface, as the total water column depth was ~20 m. Seawater was transferred from Niskin bottles into 20 L carboys (acid washed and rinsed, then rinsed three times with seawater from the sampling depth prior to filling), using silicone tubing that had been acid washed and rinsed with distilled water and then with sample water prior to use.

### Experimental setup

#### Seawater sampling and gravity filtration

Bulk (unfiltered) seawater was taken directly from carboys to measure polysaccharide hydrolase activities, peptidase activities, and glucosidase activities, and to determine the composition of microbial communities. Particle-associated communities were separated from bulk water by gravity filtration through 3 μm pore-size filter (47-mm diameter, Whatman Nuclepore), following Balmonte et al. (2018). Three replicate gravity filtration setups were used for each depth at each station to provide sufficient filters for all experiments and analyses. Water was gravity-filtered overnight either in a cold van (4°C) or in the main lab aboard ship (depending on *in situ* collection temperature, Supplementary Table S2) from a 20 l carboy, and the volume filtered was recorded (Supplementary Table S2). Filters were cut into 12 equal pieces on a sterilized glass plate using a sterile razor blade and used in measurements as described below. Filter pieces for DNA extraction (see below) were frozen at −80°C aboard ship. Note that hydrolysis rates of polysaccharides in bulk water from Stns. 1–4 have previously been reported in Hoarfrost et al. (2019).

#### Peptidase and glucosidase activities

Peptidase activities were measured using small peptides or the amino acid leucine linked to the fluorophore 7-amido-4-methyl coumarin (MCA). Chymotrypsin substrates (from BAChem) included alanine–alanine-phenylalanine (1-letter amino acid codes: AAF) and alanine–alanine-proline-phenylalanine (AAPF), and trypsin substrates (from BAChem) included glutamine-alanine-arginine (QAR) and phenylalanine-serine-arginine (FSR). Alpha- and beta-glucosidase activities were measured using α-glucose and β-glucose linked to the fluorophore 4-methylumbelliferone (MUF). This set of substrates is hydrolyzed by exo-acting (terminal-unit cleaving) enzymes, which hydrolyze α-glucose, β-glucose, and leucine, and by endo-acting (midchain-cleaving) enzymes, which hydrolyze AAF, AAPF, QAR, and FSR. We note that the trypsin and chymotrypsin substrates (AAF, AAPF, QAR, and FSR) could also potentially be hydrolyzed by exo-acting enzymes, which would have to systematically cleave each amino acid in the oligopeptide in order to free the MCA fluorophore and lead to an increase in fluorescence signal. In such a case, we would expect that the apparent rate of hydrolysis of a peptide would increase with time, since some initial time period would be required for each exo-peptidase to act successively to free the fluorophore. For our experiments, however, we measured peptidase activity every 4 h (EN584) to 6 h (EN556) for a 24 h period, and the endopeptidase substrates did not show an increase in hydrolysis rates between the earliest and latest measurement timepoints. We therefore surmise that endopeptidases, rather than successively-acting exo-peptidases, were responsible for hydrolysis of these substrates in our incubations.

Enzymatic activities in seawater were measured following Balmonte et al. (2018). In brief, the substrate was added at saturating concentrations (200 μM for EN556 and 150 μM for EN584, as determined in surface seawater at each station for EN556 and the first station of the cruise for EN584) to a total volume of 200 μl in a 96-well plate to measure the enzymatic potential of the microbial communities. For each substrate, triplicate wells of seawater amended with substrate were used in experimental incubations, and triplicate wells of autoclaved seawater amended with substrate were measured as killed controls. Fluorescence was measured immediately (t0) and every 6 h over a 24-h time period, using a plate reader (TECAN SpectraFluor Plus with 360 nm excitation, 460 nm emission for EN556; TECAN SpectraFluor Plus with 340 nm excitation, 460 nm emission for EN584). Hydrolysis rates were measured as an increase in fluorescence over time. Fluorescence signals were converted to concentrations using standard curves of MUF and MCA fluorophores. Note that hydrolysis rates of peptidases in bulk water from Stns. 1–4 have previously been reported in Hoarfrost et al. (2019).

For particle-associated communities, enzyme activities were measured by immersing one-twelfth of a filter in 7 ml autoclaved seawater. Two different filter pieces were used to measure enzymatic activities in duplicate; a sterile filter piece was used in autoclaved seawater as a killed control. Substrate was added at saturating concentrations (see above), and triplicate 200 μl subsamples were taken immediately (t0) and every 6 h over a 24-h time period. Hydrolysis rates were measured as an increase in fluorescence over time, using the plate reader as described above.

#### Polysaccharide hydrolase activities

Activities of polysaccharide-hydrolyzing enzymes were measured using six fluorescently labeled polysaccharides—pullulan, laminarin, xylan, fucoidan, arabinogalactan, and chondroitin sulfate—labeled with fluoresceinamine (Arnosti, 2003). These polysaccharides were chosen because they are found in marine phytoplankton and algae (e.g., Painter, 1983), and/or enzymes hydrolyzing these polysaccharides have been demonstrated to occur in marine bacteria (Alderkamp et al., 2007; Neumann et al., 2015).

For each substrate, three 15 ml sterile polypropylene tubes (Fischer) were filled with unfiltered seawater; one 15 ml tube filled with autoclaved seawater served as a killed control. A single substrate was added to each tube to a final concentration of 3.5 μM (except fucoidan which was added to a final concentration of 5 μM, due to its low labeling density). One tube with seawater served as a blank, and one tube filled with autoclaved seawater served as a negative control. Samples were incubated in the dark at close to *in situ* temperatures (Supplementary Table S2). For the particle-associated samples, 1/12 of a filter was incubated in autoclaved seawater to which a polysaccharide substrate was added. Due to the limited amount of filter material available, these incubations were carried out in duplicate. One-twelfth of a sterile filter incubated in autoclaved seawater served as the killed control.

To measure hydrolysis rates, subsamples were taken directly after each incubation was set up (day 0) and at five additional timepoints (2 days, 5 days, 10 days, 17 days, and 30 days). Each subsample was filtered through a 0.2 μm pore-size surfactant-free cellulose acetate filter, then stored frozen at −20°C until analysis. Hydrolysis rates were determined using gel permeation chromatography with fluorescence detection to measure the changes in the molecular weight distribution of the polysaccharides as a function of incubation time, as described in detail in Arnosti (2003). All of the enzymatic hydrolysis rates should be considered as potential rates, since the added substrate is in competition with naturally occurring organic matter for enzyme active sites.

### Bacterial community analysis

For bulk water samples, 2 to 3 l of seawater was filtered through a 0.2 μm pore-size 47 mm diameter Whatman Nuclepore track-etched Membrane filter, and stored at −80°C until DNA extraction. For the bulk samples, DNA was extracted from a quarter of each filter, which was cut using a sterile razor blade or sterile scissors. For the PA samples, one slice of the 3 μm filters was used for DNA extraction. All DNA was extracted using a DNeasy PowerSoil Kit (Qiagen). For select samples, duplicate extractions were performed to determine the accuracy of extraction and sequencing. DNA libraries were prepared using the Nextera XT Index Kit, v2 set A (Illumina) according to manufacturer protocol. The V1-V2 hypervariable region of the 16S rRNA gene was amplified using the primers 8F (5′- AGA GTT TGA TCC TGG CTC AG-3′) and 338R (5′- GC TGC CTC CCG TAG GAG T-3′) (custom made by Integrated DNA Technologies; Balmonte et al., 2018) with the Illumina-specific forward primer overhang adapter (5′-TCG TCG GCA GCG TCA GAT GTG TAT AAG AGA CAG-3′) and reverse overhang adapter (GTC TCG TGG GCT CGG AGA TGT GTA TAA GAG ACA G), and sequenced at the UNC High-Throughput Sequencing Facility using Illumina MiSeq PE 2×250.

Sequenced multiplexed paired-end FASTQ files were imported into QIIME2 (version 2019.4^1^; Bolyen et al., 2019), demultiplexed, and then denoised and dereplicated using DADA2 (Callahan et al., 2016). Quality control was performed during the DADA2 denoising and dereplicating process, with a Phred quality control cutoff of 25 (i.e., 10 bases) filtered prior to merging of paired-end reads. A Naïve Bayes classifier was trained to assign taxonomy to ASVs using reference sequences from the Silva 16S rRNA database (version 132; Pruesse et al., 2007) sequenced with the 8F and 338R primers.

The BIOM-formatted OTU table and phylogenetic tree of representative sequences were imported into R; the phyloseq package (version 1.32.0; McMurdie and Holmes, 2013) was used to remove chloroplasts and rarefy samples to an even sampling depth of 30,000 sequences per sample to enable comparison of bacterial relative proportions across samples with initially uneven sequencing depths. Bubble plots of community composition were filtered at the 5% level for the Class-level plot and the 3% level for the Family-level plot.

### Statistical analyses

To visualize depth, station, and size fraction patterns of enzymatic activities, Bray–Curtis dissimilarity indices were calculated for each size fraction and were ordinated using Nonmetric multidimensional scaling (NMDS). To explore differences in bacterial community composition, Bray–Curtis dissimilarity indices were calculated for each community and were ordinated using NMDS. The NMDS plots were ordinated from the provided OTUs after filtering out chloroplasts and rarefying samples to an even sampling depth; no further filtering was carried out. Permutational multivariate analysis of variance (PerMANOVA) was used to quantify the variation in enzymatic rates and community dissimilarities (Bray–Curtis) as a function of water depth, size fraction, and station. Standard *t*-tests (two-tailed, unpaired, and heteroscedastic) were used to make direct comparisons for enzymatic activities between depths and locations (coastal vs. offshore). To distinguish the breadth/spectrum of activities measured between the PA and bulk water samples, the number of substrates hydrolyzed at each station/depth was added. For instance, in surface waters at Stn. 5, six polysaccharides were hydrolyzed in the PA fraction while only three were hydrolyzed in the bulk water samples. These values were log transformed, and a *t*-test was used to compare the spectrum of activities between the PA fraction and the bulk water. Corrplot (version 0.92), based on Pearson’s correlations, was used to visualize correlations between enzymatic activities.

## Results

### Water mass characteristics

Epipelagic water from the majority of the stations (Stns. 5, 8, 13, 14, 15, and 16) had T/S characteristics typical of North Atlantic Surface Water, or a mixture of Gulf Stream Water and North Atlantic Surface Water (Stns. 10, 11, 12, and 12r; Supplementary Table S1; Supplementary Figure S2). Water masses at Stns. 4 and 9 differed considerably, due to the inshore location of Stn. 9 (water column depth: 20 m), and the shelf break location of Stn. 4 (water column depth: 200 m). Surface water at Stn. 9 was characteristic of surficial coastal waters (27°C; 35 PSU); Stn. 4 had notably cold shelf water at the surface (8° C; 33.5 PSU) overlying bottom water originating from a subducted warm core ring (10°C; 35 PSU; Hoarfrost et al., 2019) and thus had characteristics similar to surface water at Stn. 8 (Balmonte et al., 2019). For Stns. 10–16, water at the DCM and 300 m is characteristic of Subtropical Underwater/North Atlantic Central Water, and water at the OMZ and 1,500 m is characteristic of Intermediate Water and North Atlantic Deep Water, respectively (Heiderich and Todd, 2020).

### Patterns of peptidase and glucosidase activities

**Figure 1:**
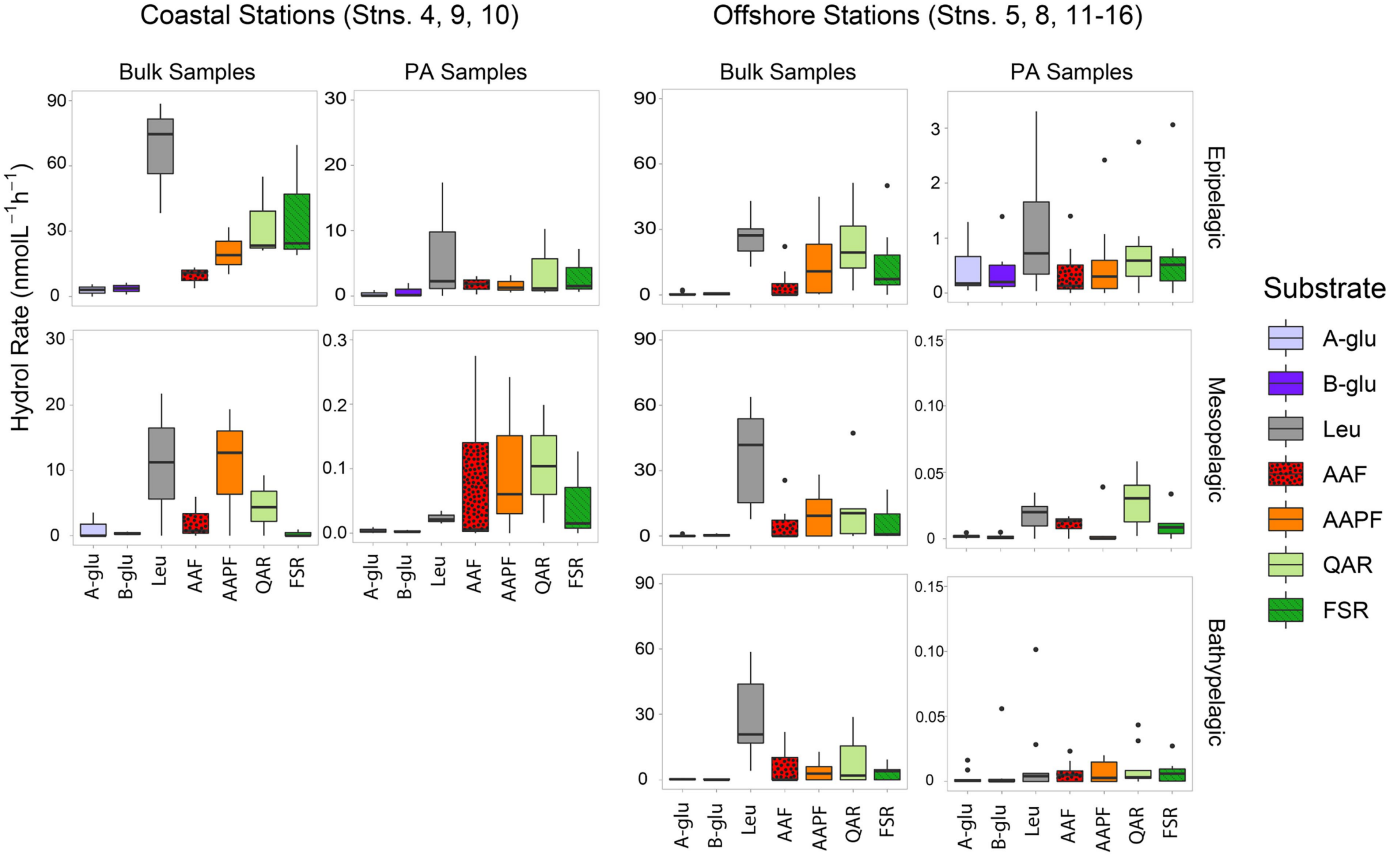
Box and whisker plot of glucosidase and peptidase activities of bulk and particle-associated (PA) water samples; line in middle represents median value, top and bottom of the box represent the upper and lower quartile, respectively, ends of ‘whiskers’ represent lowest and highest values (dots: outliers). Coastal stations include Stns. 4, 9, and 10, and offshore stations include Stns. 5, 8, and 11–16. A-glu, alpha-glucosidase; B-glu, beta-glucosidase; Leu, leucine aminopeptidase; AAF, alanine–alanine-phenylalanine-chymotrypsin; AAPF, alanine–alanine-proline-phenylalanine-chymotrypsin; QAR, glutamine-alanine-arginine-trypsin; FSR, phenylalanine-serine-arginine-trypsin. Note that the y-axis differs for bulk and PA samples at each depth.

Peptidase and glucosidase activities in bulk water showed distinct depth and spatial patterns. Peptidase activities in coastal epipelagic waters were considerably higher (activities ranging from ~10–75 nmol L^−1^ h^−1^) than at offshore locations (activities from ~2–28 nmol L^−1^ h^−1^; *p* = 0.0489; Figure 1). Subsurface activities in coastal stations were lower than activities in epipelagic water, whereas at offshore stations, activities in epipelagic waters, the upper mesopelagic, and bathypelagic waters differed less from one another (Figure 1). Notably, there was a significant difference in enzymatic activity between mesopelagic and epipelagic waters at coastal stations (Figure 1; *p* = 0.000343), while the difference between enzyme activities of these waters offshore was not significant (Figure 1; *p* = 0.4715). Overall, the exo-acting leucine aminopeptidase contributed the most to summed activities in coastal epipelagic waters; offshore, exo-and endopeptidase activities were generally comparable in epipelagic waters, but leucine aminopeptidase activities were higher than endopeptidase activities at depth, often contributing more than 50% to the total summed activities (Figures 1; Supplementary Figure S3).

Throughout the water column, particle-associated bacteria hydrolyzed a broader spectrum of glucosidase and peptidase substrates compared to bacteria in bulk water (Figure 1; Supplementary Figure S3; *p* = 0.036). In offshore surface waters, in particular, α- and β-glucosidase activities contributed considerably to PA activities, a pattern not seen in the bulk water samples (Figure 1). The contribution of α- and β-glucosidase activities to PA coastal samples was considerably lower relative to the offshore PA samples (Figure 1; Supplementary Figure S3). In meso- and bathypelagic waters offshore, endo-peptidase activities dominated, often making up more than 75% of the total summed activities (Supplementary Figure S3), in contrast to leucine aminopeptidase activity dominating bulk waters.

Scaled by the volume of water filtered to obtain particles, the contribution of PA activities relative to bulk activities decreased with depth as well as with distance from shore. In coastal epipelagic water, the PA activities were generally *ca.* 20% of the bulk activities, but this contribution dropped to *ca.* 1% of the bulk activities at subsurface coastal depths (Figure 1). Offshore, PA activities constituted *ca.* 1% of bulk activities in epipelagic waters and dropped by several orders of magnitude with increasing depth, due in part to the higher amounts of water filtered *via* gravity filtration (and the likely correspondingly lower concentrations of particles) with increasing depth (Supplementary Table S2).

**Figure 2:**
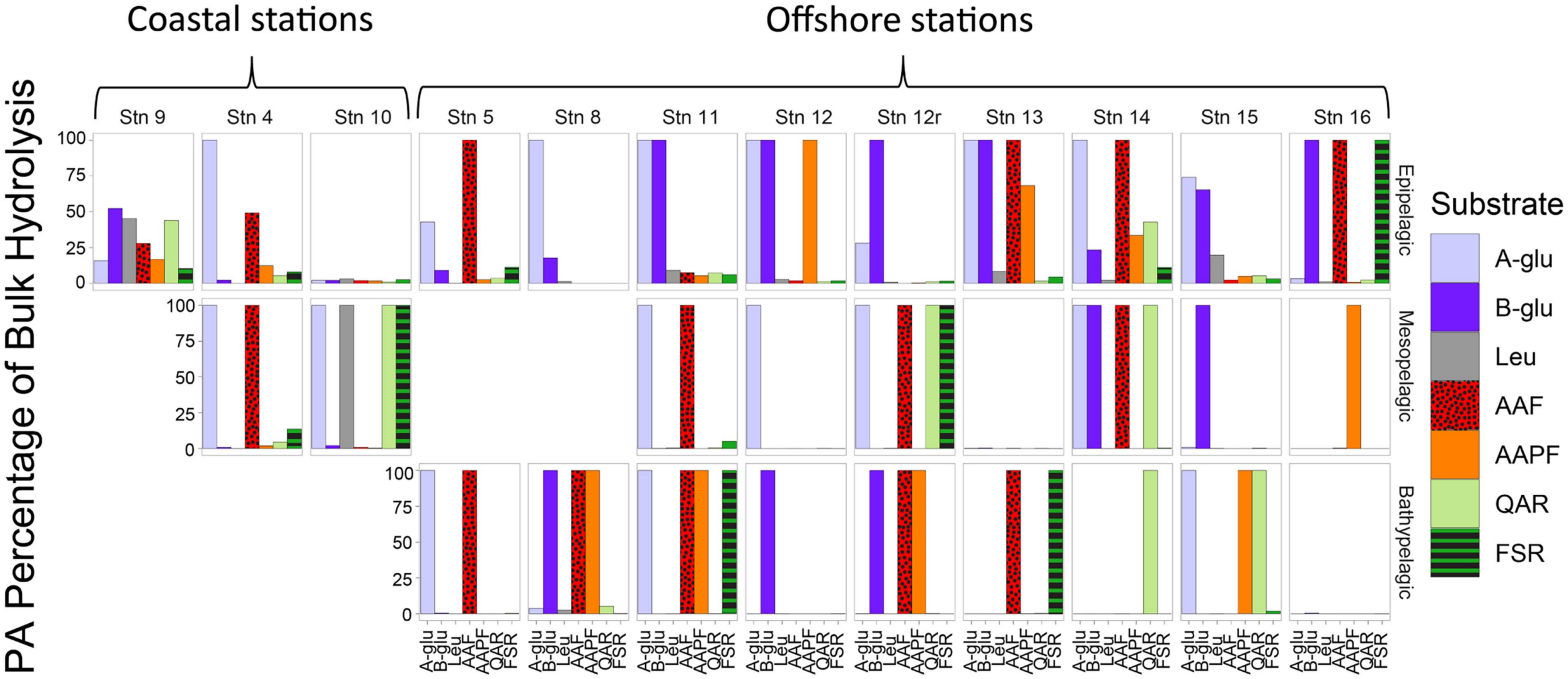
Percent of bulk water glucosidase and peptidase activities that is attributable to the PA fraction, calculated by comparing the maximum rate of the bulk water and PA samples. A-glu, alpha-glucosidase; B-glu, beta-glucosidase; Leu, leucine aminopeptidase; AAF, alanine–alanine-phenylalanine-chymotrypsin; AAPF, alanine–alanine-proline-phenylalanine-chymotrypsin; QAR, glutamine-alanine-arginine-trypsin; FSR, phenylalanine-serine-arginine-trypsin.

Although PA activities were only a small fraction of the total bulk water activities, at almost all depths and stations, specific activities were only measurable in the PA samples (Figure 2). With the sole exception of the epipelagic waters at Stns. 9, 10, and 15, and bottom waters at Stn. 16, one or more of the activities measured was measurable only in the PA fraction. Moreover, at most of the stations and depths, two or more activities were measurable only in the PA fraction. The specific activities measured only in the PA fractions varied somewhat by station and depth, but glucosidases (α- and β-) and chymotrypsin activities (AAF and AAPF) commonly belonged to this category; trypsin activities were also occasionally measured only in the PA fraction.

### Patterns of polysaccharide hydrolase activities

**Figure 3:**
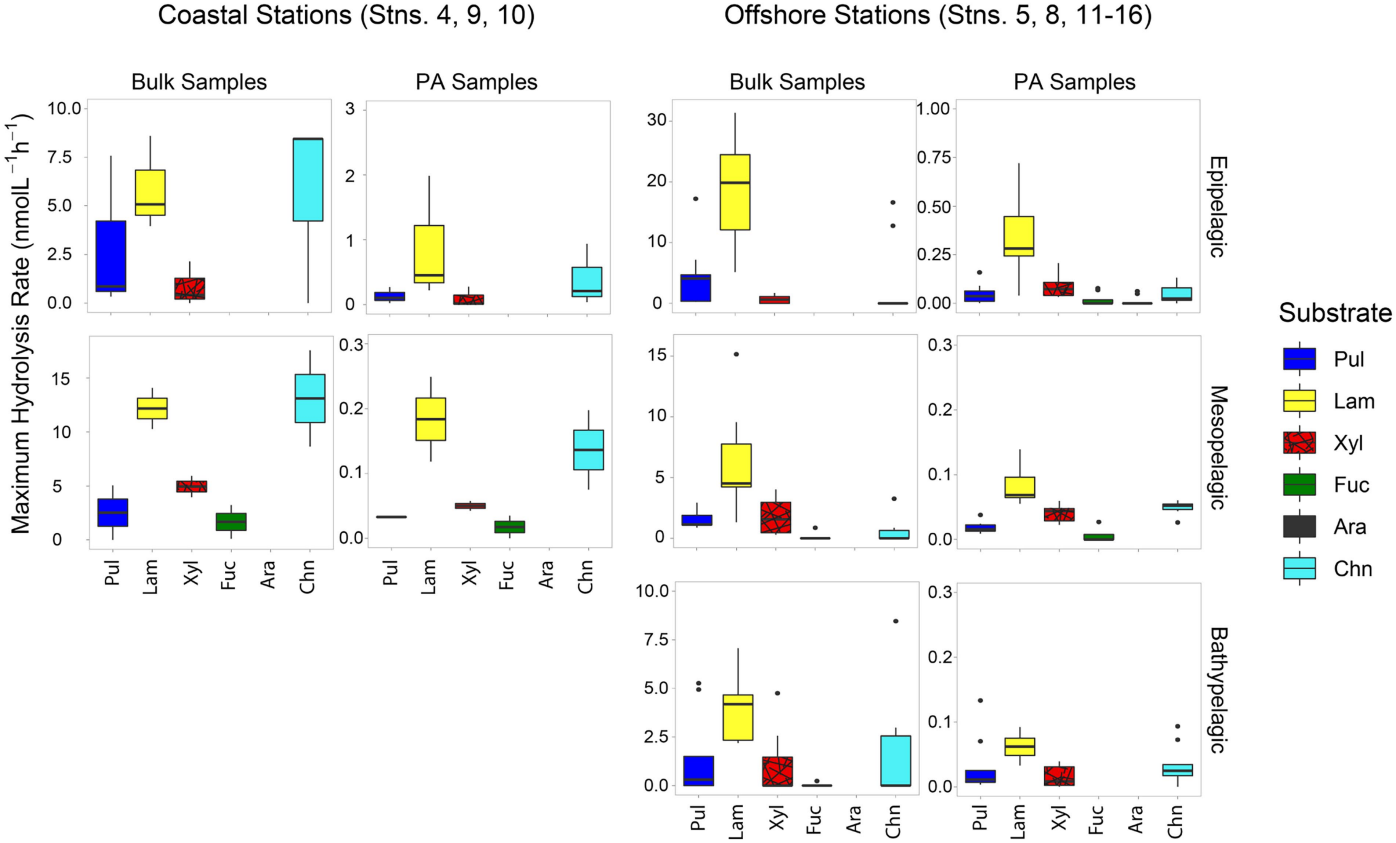
Box and whisker plot of maximum polysaccharide hydrolase activities of bulk and PA water samples; line in middle represents median value, top and bottom of the box represents the upper and lower quartile, respectively, ends of ‘whiskers’ represent lowest and highest values (dots: outliers). Coastal stations include Stns. 4, 9, and 10, and offshore stations include Stns. 5, 8, and 11–16. Note that the y-axis differs for bulk and PA samples at each depth. Pul, pullulan; Lam, laminarin; Xyl, xylan; Fuc, fucoidan; Ara, arabinogalactan; Chn, chondroitin sulfate.

Patterns of polysaccharide hydrolase activities also varied by location and depth. In all bulk water samples, only a limited spectrum of substrates was hydrolyzed; none of the bulk water samples showed hydrolysis of all six polysaccharides (Figure 3). The spectrum of substrates hydrolyzed in coastal stations was somewhat broader (a maximum of four substrates hydrolyzed) than at offshore stations (a maximum of three substrates; Figure 3; Supplementary Figure S4). Laminarinase was the only activity detected at all sites and depths, and was usually the highest activity measured at offshore stations. Although laminarinase activity was highest in epipelagic waters and lower in mesopelagic and bathypelagic waters, the depth-related decrease in hydrolysis rates in bulk waters of offshore stations was modest. This trend did not hold for all polysaccharide hydrolase activities, since chondroitin hydrolysis was detectable more frequently in meso- and bathypelagic than in epipelagic offshore waters.

A much broader spectrum of polysaccharide hydrolase activities was measurable in the PA fraction compared to the bulk water samples (Figure 3; *p* = 0.00011). All six substrates were hydrolyzed in the PA fraction of surface waters from Stn. 5, and typically four or five substrates were hydrolyzed in the other PA samples, compared to two or three substrates for most bulk samples (Supplementary Figure S4). Although a broader spectrum of activities was detected in the PA fraction, laminarinase activity still dominated on particles (Figure 3) and generally was higher than for other substrates (Supplementary Figure S4).

**Figure 4:**
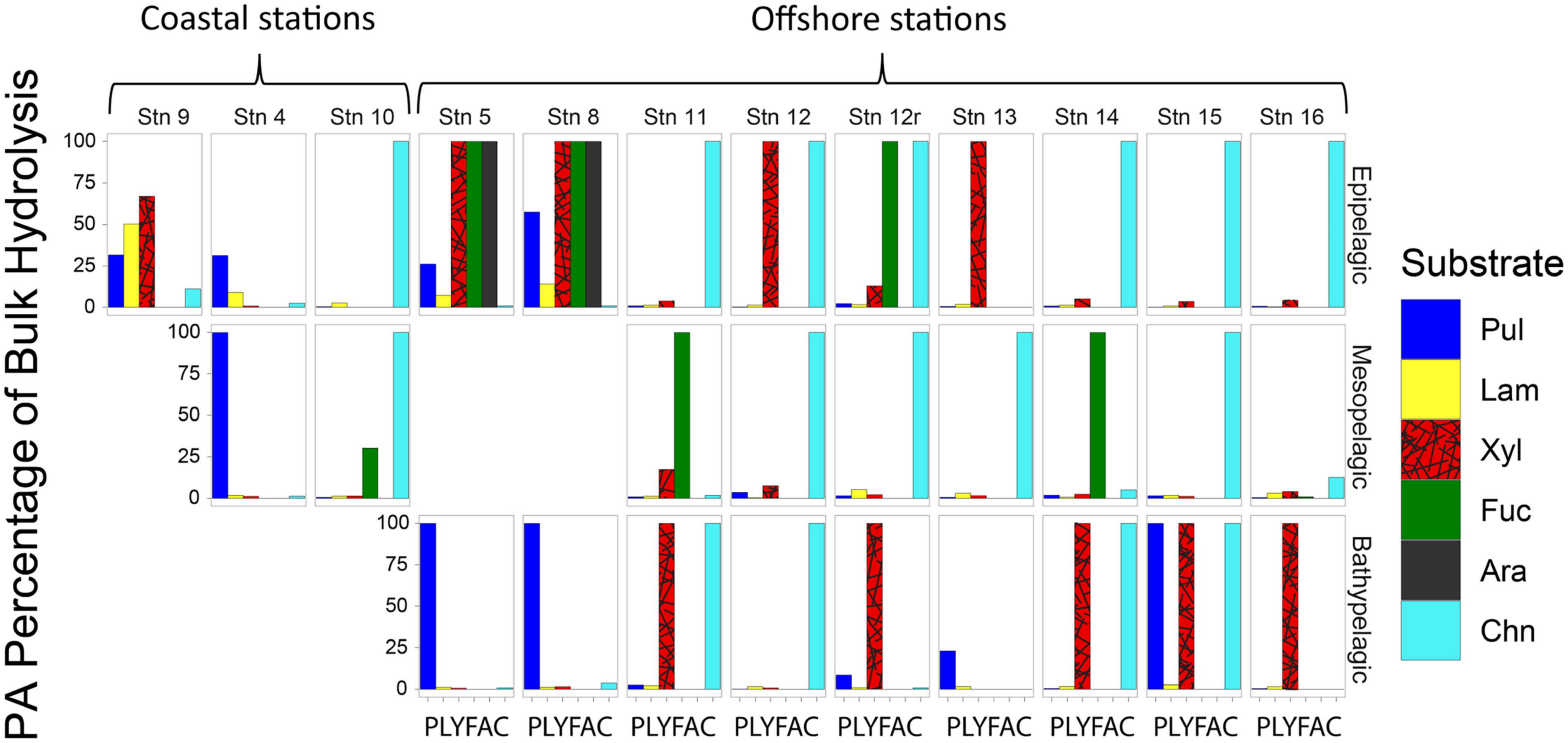
Fraction of bulk water polysaccharide hydrolase activities that is attributable to the PA fraction at each station and depth, calculated by comparing the maximum rate of the bulk water and the PA samples. Pul, pullulan; Lam, laminarin; Xyl, xylan; Fuc, fucoidan; Ara, arabinogalactan; Chn, chondroitin sulfate.

Although—calculated on a volume-equivalent basis—PA polysaccharide hydrolase activities were typically only a fraction of the activities measured in bulk water, for most stations and depths, a number of activities were measurable only in the PA fraction, thereby accounting for 100% of that specific activity (Figure 4). Moreover, the PA polysaccharide hydrolase activities decreased only moderately with depth (by a factor of 6–10) offshore (Figure 3), a much smaller decrease compared to the decrease in peptidase and glucosidase activities over the same depth range (Figure 1).

### Bacterial community composition

**Figure 5:**
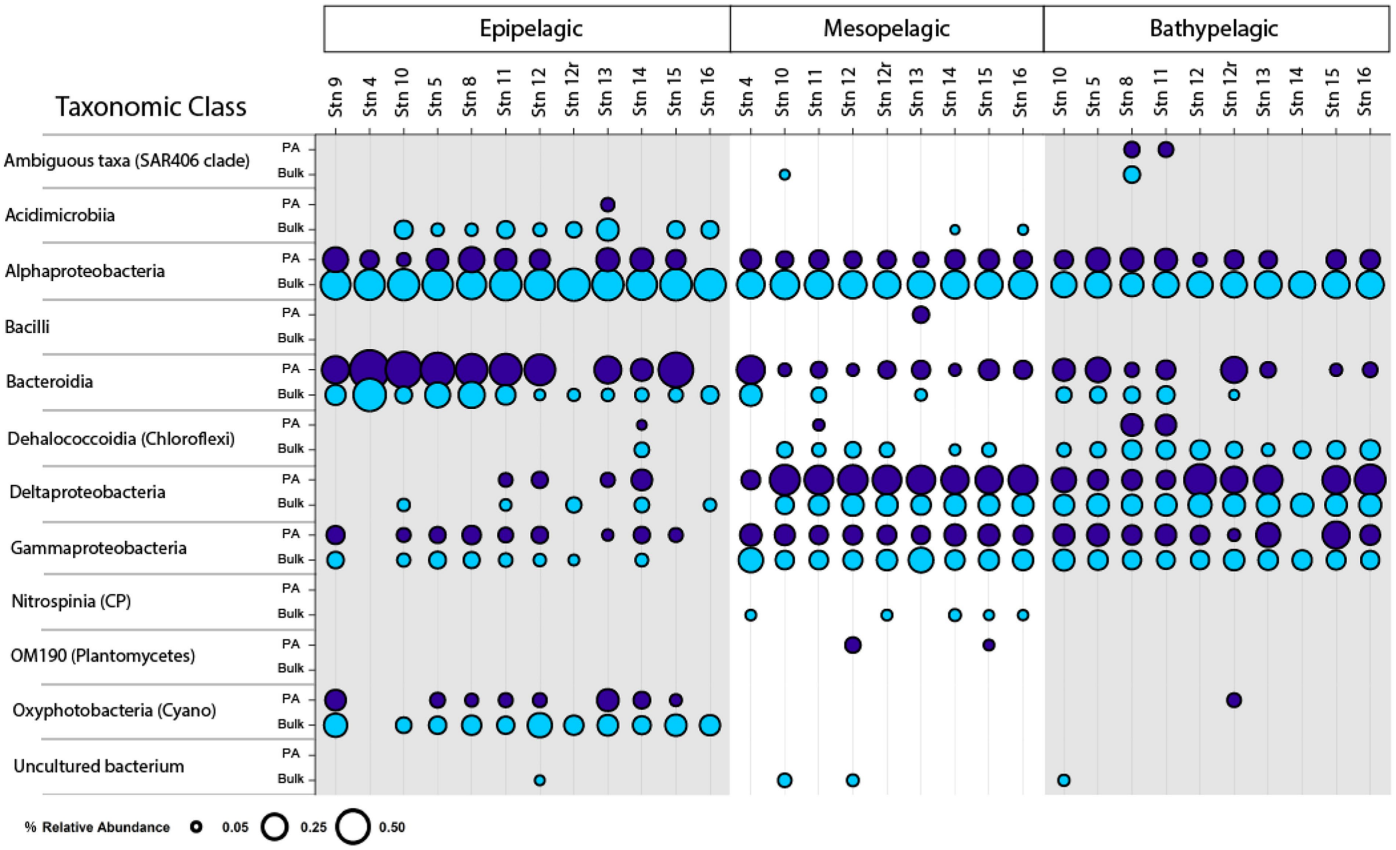
Class-level composition of bulk (unfiltered) and PA bacterial communities. Circle size correlates with relative abundance. Data shown were filtered and represents classes with ≥5% relative abundance in the data set.

Bacterial community composition, as represented by relative read abundance, differed notably with depth (Figure 5, PerMANOVA, *p* = 0.001, *R*^2^ = 0.25291). At the class level, epipelagic water communities were typically represented by Bacteroidia, Oxyphotobacteria (Cyanobacteria), and Alphaproteobacteria. In the mesopelagic zone, Deltaproteobacteria, Alphaproteobacteria, and Gammaproteobacteria tended to dominate. In bathypelagic waters, there was a more even distribution of bacteria from Alphaproteobacteria, Bacteroidia, Dehalococcoidia, Deltaproteobacteria, and Gammaproteobacteria (Figure 5). Both Deltaproteobacteria and Gammaproteobacteria showed increases in relative abundance with increasing depth, while Bacteroidia typically decreased in relative abundance with increasing depth (Figure 5). PA and bulk communities also differed notably (PerMANOVA, *p* = 0.001, *R*^2^ = 0.11534). Bacteroidia (and to a large extent, Deltaproteobacteria) were more abundant in the particle-attached communities, whereas Alphaproteobacteria were more abundant in the bulk bacterial communities.

At the family level, there were also differences between bacterial communities, associated with depth, shelf location, and particle-association (Supplementary Figure S5). Relative read abundance of Flavobacteriaceae and NS9 marine group (Bacteroidia) decreased with depth, while the relative read abundance of the Thioglobaceae and Alteromonadaceae (Gammaproteobacteria), Bdellovibrionaceae and other uncultured members of the Deltaproteobacteria, and Nitrospinaceae (Nitrospinia) increased with increasing depth. Comparing the PA and bulk communities, Flavobacteriaceae, the NS9 marine group, and Alteromonadaceae were more abundant in the PA communities, whereas members of the alphaproteobacterial Clade I and Clade II (SAR11 clades) and Rhodobacteraceae were relatively more abundant in the bulk bacterial communities.

### Analyses of patterns of community composition and function

**Figure 6:**
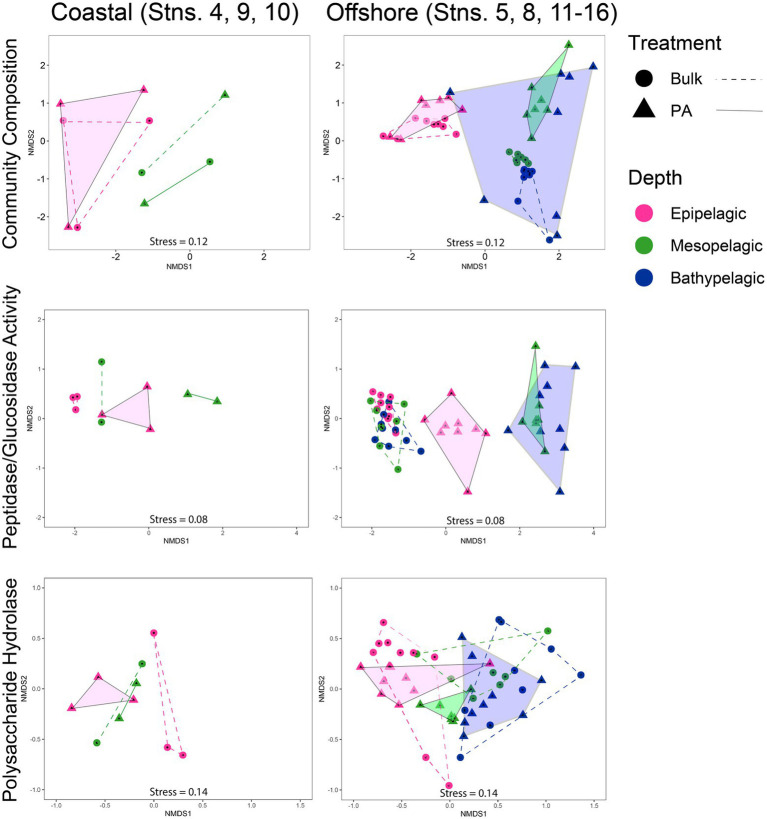
Non-metric multidimensional scaling (NMDS) of community composition, glucosidase and peptidase activities, and polysaccharide hydrolase activities, separated into coastal and offshore stations using Bray–Curtis dissimilarity index. PA data are represented by triangles and are outlined and shaded; bulk data are represented by circles and are outlined with a dashed line.

Overall, the composition of PA and bulk bacterial communities was more similar in epipelagic waters, but became more dissimilar at deeper depths, as shown by non-metric multidimensional scaling (NMDS) plots, based on Bray–Curtis dissimilarity (Supplementary Figure S6). Separating the coastal from the offshore stations and focusing on the offshore communities shows that PA community composition was most dissimilar among the bathypelagic stations (Figure 6). The mesopelagic PA communities were less widely separated from one another, but were distinct from the more-tightly clustered mesopelagic bulk water communities; epipelagic PA communities overlapped with the bulk communities. Analysis of the offshore peptidase and glucosidase activities showed distinct separation of the bulk from the PA fraction; within the PA fraction, peptidase and glucosidase activities in epipelagic waters separate from those in mesopelagic and bathypelagic waters (Figure 6). In contrast, the peptidase and glucosidase activities for the bulk communities of all three depths clustered closely together. The PA polysaccharide hydrolase activities also separated by depth, with complete separation between epipelagic and mesopelagic waters, and near-complete separation between mesopelagic and bathypelagic waters; for bulk waters, there is complete separation between epipelagic and bathypelagic activities, but overlap between mesopelagic and bathypelagic activities measured (Figure 6).

## Discussion

### Selected enzyme activities are detectable only in the PA fraction

Particle-associated microbial communities may play a disproportionately large role in the production of specific enzymes involved in organic matter degradation (Figures 2, 4). Although some enzyme activities were not detectable in measurements of bulk waters, they were measured in the particle-associated samples. This difference in the range of enzymes detectable in the PA vs. in the bulk water may be due to the cost/benefit balance of enzyme production (e.g., Vetter et al., 1998), considering the higher concentration of cells and organic matter on particles. Hydrolysis of complex particulate organic matter may require an array of extracellular enzymes, but the effort may be shared by the greater number of cells found on particles, and therefore can pay off from an energetic perspective (Allison, 2005). Such a concentration of cells can in fact trigger production of specific enzymes through mechanisms such as quorum sensing (Hmelo et al., 2011; Jatt et al., 2015; Krupke et al., 2016), which leads to bacteria ‘sharing’ the hydrolysis products resulting from the enzymes that they collectively produce. Although our experimental approach may have enhanced this response by concentrating particles and cells, or by providing additional surface area (the filter piece) for cell attachment and growth, in the environment, a similar response can occur through formation of aggregates (Ziervogel et al., 2010), which have been shown to be hotspots of enzymatic activity (Grossart et al., 2003a,b; Prairie et al., 2015).

We recognize that the PA fraction was isolated from the bulk water, and therefore constitutes a portion of the bulk water. Isolating the PA fraction in order to separately investigate the enzymatic activities of this fraction in effect provides a ‘magnifying glass’ focused on their specific enzymatic capabilities. These observations do not exclude the possibility that some of the enzyme activities measured on the PA fraction may also have been present among non-particle-associated members of the bulk water fraction. In such a case, however, they were below our limit of detection, and thus must have been present in a small fraction of bacteria, or have been carried out by enzymes with low turnover numbers. Our interpretation that the substantial difference in enzymatic capabilities of PA compared to bulk water incubations—particularly offshore—is due to inherent differences among the microbial communities, rather than simply being a function of cell numbers, is supported by the distinct difference in the composition of PA and bulk water communities that closely paralleled differences in enzymatic function (Figure 6). The PA communities were not simply highly amplified replicates of the bulk water communities. This interpretation is also supported by comparisons of the genomic potential of PA and free-living bacterial communities that have documented much greater potential to produce extracellular enzymes and consume complex high molecular weight substrates among the PA compared to free-living fractions (Zhao et al., 2020; Leu et al., 2022). The higher relative abundance of Bacteroidia in the PA fraction at all depths (Figure 5) also supports this interpretation, since members of the Bacteroidia are well-known for their role in producing extracellular enzymes used to degrade complex organic matter (Lapebie et al., 2019).

The more pronounced difference between PA and bulk water enzymatic capabilities in offshore relative to coastal environments (Figure 6) is likely due in part to lower particle loads in offshore environments. In contrast, bulk coastal waters, especially at Stns. 4 and 9, where the gravity filtration volume was much lower due to high particle content (Supplementary Table S2), likely carry a much stronger PA imprint. Moreover, higher particle content in coastal waters may lead to considerably more exchange between PA and free-living bacteria (Yawata et al., 2020), and thus to fewer differences in enzyme activities between the bulk water and the PA fraction (Figures 1, 3).

### Depth-related distinctions in community composition and function

Bulk epipelagic and meso−/bathypelagic communities differed distinctly in composition (Figure 6), in accordance with reports from other locations in the ocean (Mende et al., 2017; Mestre et al., 2018; Boeuf et al., 2019). These differences in the western North Atlantic, evident also for PA communities, are likely driven in part by the high relative read abundance of Cyanobacteria in epipelagic waters compared to deeper depths (Figure 5). However, neither the increasing dissimilarity among PA communities with depth (Figure 6), nor the functional differences among these communities (Figure 6), are explained by the presence or absence of Cyanobacteria. Given that the bulk communities also include the bacteria collected on particles, the greater dissimilarity of the PA communities compared to bulk communities–especially in the bathypelagic ocean–indicates that gravity filtration effectively concentrated a numerically less-abundant fraction of the total community, including members with distinct identities and functions.

This concentration of a select portion of the bacterial community may in part be the outcome of optimal foraging considered in terms of patch use theory (Yawata et al., 2020). This theory suggests that particle detachment by bacteria is more likely in resource-rich environments, since the probability of a bacterium finding another richer resource patch is high. In resource-rich environments, the composition of the bulk and PA communities would more closely resemble one another, due to this frequent detachment and reattachment process. This observation is consistent with our data from coastal environments (Figure 6). Conversely, optimal foraging theory suggests that detachment becomes less likely as particles and the surrounding environment become more resource-poor, thus leading bacteria that are suited to degrade particulate organic matter to remain attached once they have located a particle in a particle-poor environment (Yawata et al., 2020). Numerically less-abundant bacteria – members of the ‘rare biosphere’ (Sogin et al., 2006) – could thereby become concentrated on particles in the deep ocean. Such bacteria likely have characteristics such as enhanced abilities to produce specific extracellular enzymes that lead to their preferential enrichment on particles (Lambert et al., 2019).

We observed a relative enrichment of both Bacteroidetes and of Deltaproteobacteria in PA samples, a pattern very similar to PA enrichments of Flavobacteriia (Bacteroidota) and of Deltaproteobacteria reported for PA bacterial communities from western North Atlantic (Zorz et al., 2019). The increased relative abundance of Bacteroidetes in PA compared to bulk communities (Figure 5) may be an example of specific enrichment related to particle resources and metabolic capabilities, since members of the Bacteroidetes are known to produce a very wide range of polysaccharide- and protein-hydrolyzing enzymes (Xing et al., 2015; Krüger et al., 2019; Lapebie et al., 2019) that are required to access high molecular weight substrates.

Variability in particle source, especially offshore, may additionally account for some of the patterns of bacterial communities and activities that we observe. POM in the ocean is diverse in origin and composition, including fecal pellets, marine snow, and aggregates, comprising suspended as well as sinking POM (Nagata et al., 2010; Bochdansky et al., 2016). Sinking particles also undergo considerable compositional change during their transit to deeper depths in the ocean (Wakeham et al., 1997). The greater variation among PA community composition measured in the deeper samples thus may be due to processes including colonization by bacteria of different particle sources in the upper ocean (Mestre et al., 2018), succession of microbial communities as particles sink and organic matter is utilized by specific organisms (Fontanez et al., 2015; Thiele et al., 2015; Valencia et al., 2022), and differences in the ability of a range of organisms to grow on sinking vs. suspended particles (Baumas et al., 2021). At deeper depths, the difference between PA and free-living bacterial communities is larger (Salazar et al., 2015; Ruiz-González et al., 2020; Leu et al., 2022), which could in part account for dispersion of bacterial communities with depth.

Much of the POM that reaches the bathypelagic ocean may in fact be metabolized by microbial communities found at depth, rather than by the communities initially attached to POM in the upper water column (reviewed in Nagata et al., 2010; Tamburini et al., 2013), such that different functional capabilities are a requirement to access deep ocean POM. Support for this perspective is provided by differences in composition and metabolic potential of microbial communities of the surface and bathyal/abyssal ocean (DeLong et al., 2006; Leu et al., 2022). Additional support is provided by the observation that sinking POM collected by sediment traps at bathypelagic depths is colonized by microbes typical of the bathypelagic ocean (Preston et al., 2020; Poff et al., 2021). Bacterial communities of the meso- and bathypelagic ocean have also been found to have a higher abundance of genes associated with excreted (extracellular) peptidase and carbohydrate hydrolases compared to their epipelagic counterparts, thus indicating their potential to enzymatically access complex organic substrates (Zhao et al., 2020).

In the western North Atlantic, the broad-scale differences in enzymatic function with depth—especially among peptidases and glucosidases—mirror the picture of bacterial community composition. The PA activities at individual stations were separated more distinctly by depth than the bulk activities, and the meso/bathypelagic samples were distinctly different from epipelagic PA samples (Figure 6). A similar pattern of increasing divergence of PA peptidase and glucosidase activities with increasing depth has also been observed along a latitudinal transect in the Pacific Ocean (Balmonte et al., 2021), suggesting that this functional separation may be a feature of open ocean environments. While global-scale investigations of microbial community composition in the bathypelagic have highlighted the distinctions of PA communities between different oceanic basins and water masses (Salazar et al., 2016), our data suggest that even within the same water masses (Supplementary Figure S2), the specific functions of PA communities as well as the composition of those communities may vary considerably (Figure 6).

### Particles as ‘specialty centers’ of degradation across the ocean continuum

**Figure 7:**
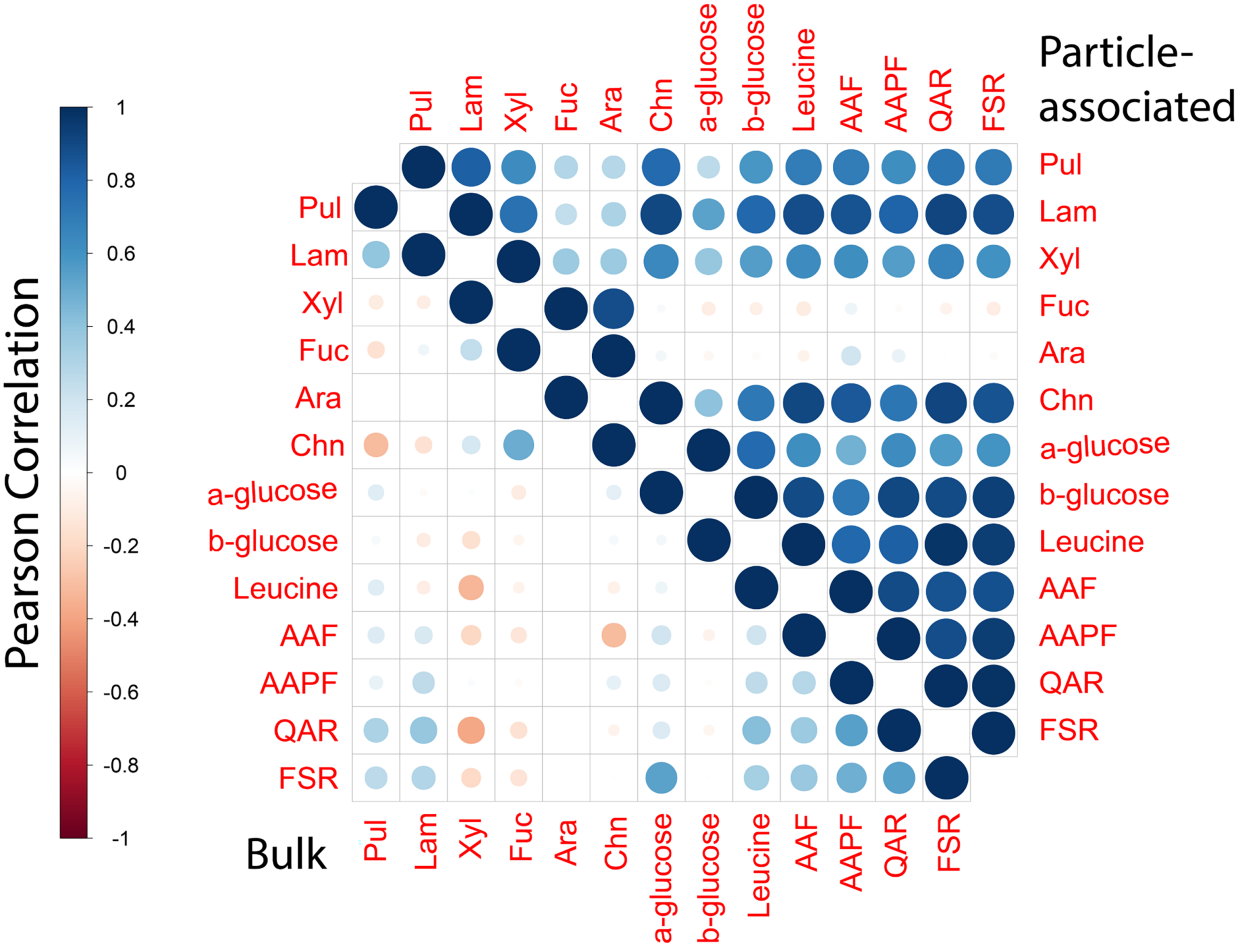
Correlation plot displaying the Pearson’s correlation between different enzymatic activity measurements for bulk and PA samples. Blue denotes positive correlation while red denotes negative correlations. The size of the circle as well as the shade of the color emphasizes the extent of correlations between different enzymatic activities.

Polysaccharide hydrolase and peptidase activities of PA bacteria show many positive correlations with one another (Figure 7), despite the fact that these two classes of enzymes target fundamentally different macromolecules. Moreover, diversity among polysaccharide hydrolases is extremely high (Laine, 1994). The PA bacterial community likely is comprised a broad range of bacteria with different enzymatic complements. The only two PA activities that show few correlations (except to each other; Figure 7) are for hydrolysis of arabinogalactan, and fucoidan, complex polysaccharides whose hydrolysis is comparatively rarely measurable in marine waters (Arnosti et al., 2011). Fucoidan in particular is structurally complex: its hydrolysis requires a very large suite of enzymes (Sichert et al., 2020); the hydrolysis of fucoidan and of arabinogalactan thus is likely carried out by entirely different organisms, using different sets of enzymes. These activities may be connected statistically in that they are not frequently measurable (Figure 4).

In comparison to activities of the PA community, the enzymatic activities of the bulk community showed comparatively few correlations with each other, either positive or negative. This general dearth of correlations among the bulk water activities highlights the variability of their enzymatic complements, seen previously from patchiness in observed peptidase and polysaccharide hydrolase activities (Balmonte et al., 2021). The pattern of high correlations among widely divergent enzymes of the PA communities is evident even when analyzing activities separately by depth (Supplementary Figure S7). Such correlations point directly to the breadth of enzymatic tools expressed by these communities, and likely hint at their differences from the bulk community in their energetic cost–benefit balances for enzyme production (Vetter et al., 1998).

**Figure 8:**
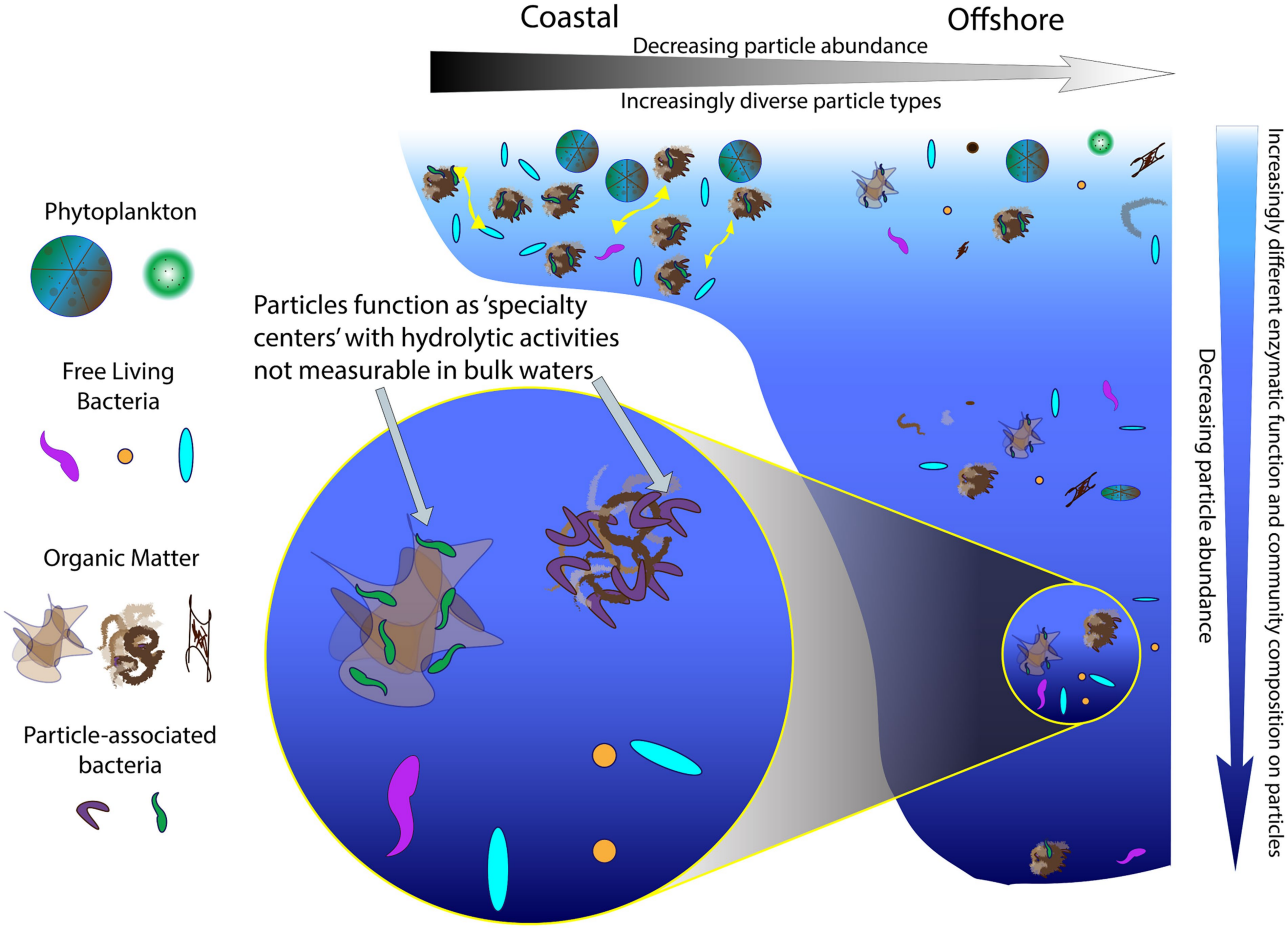
A continuum of particles and enzyme activities in the ocean. Particles serve as ‘specialty centers’ of enzymatic activities, showing an increase in functional capabilities that differs with location (coastal vs. offshore) and with depth in the ocean.

In sum, particles act as ‘specialty centers’ (Figure 8), where communities with distinct identities produce diverse enzymes that can dismantle complex organic matter, the fuel for much of the heterotrophic activity in the deep ocean (Hansell et al., 2009). The importance of PA communities in organic matter degradation has previously been recognized (e.g., Smith et al., 1992; Azam and Long, 2001), but prior studies have examined activities of only a limited range of enzymes (Grossart et al., 2003a,b; Kellogg and Deming, 2014) or have focused on locations in the upper ocean (D’Ambrosio et al., 2014). Our study demonstrates that also in the bathypelagic ocean—including depths at 1500 m and below—PA communities can produce a diverse array of enzymes whose activities are not detected in bulk waters (Figures 2, 4). To the best of our knowledge, these measurements of PA polysaccharide hydrolase activities are the only measurements from the bathypelagic ocean, at depths exceeding the 1,000 m depths measured by Balmonte et al. (2021).

## Conclusion

Particle-associated communities and their enzyme activities can be visualized as a continuum (Figure 8), characterized by decreasing particle abundance and increasing particle dissimilarity from coastal to offshore environments. With increasing depth, particle abundance also decreases (Turner, 2015), as PA community composition and enzymatic capabilities become increasingly distinct from their bulk water counterparts (Figures 6, 7). In part, this distinction may be due to the highly specialized nature of the enzymatic functions that are required to access the complex organic matter, which is increasingly difficult to characterize chemically at deeper depths (Wakeham et al., 1997; Kharbush et al., 2020). Such specialized enzymatic functions are also less likely to be redundant among community members (Martiny et al., 2013; Gralka et al., 2020), so a wider range of bacteria are required to hydrolyze these complex substrates. This greater breadth of bacteria with distinctly different enzymatic tools may underlie the increasingly dissimilar composition of PA communities at depth. Thus, PA bacteria, with a broad range of enzymatic capabilities, play a role disproportionate to their relative numbers (Lambert et al., 2019) in degrading complex organic matter in the ocean.

## Data availability statement

Raw sequence files are available on NCBI Sequence Read Archive under the accession number PRJNA 816842 (https://www.ncbi.nlm.nih.gov/bioproject/PRJNA 816842).

## Author contributions

JB, AH, and CA designed the study. SB, JB, AH, SG, and CA conducted the fieldwork and sample collection. All authors helped with processing the samples. CL analyzed the enzymatic samples. CL and SB analyzed the biological data. CL and CA wrote the manuscript with input from all co-authors. All authors contributed to the article and approved the submitted version.

## Funding

This study was funded by the U.S. National Science Foundation (OCE-1332881, OCE-1736772, and OCE-2022952 to CA).

## Conflict of interest

The authors declare that the research was conducted in the absence of any commercial or financial relationships that could be construed as a potential conflict of interest.

## Publisher’s note

All claims expressed in this article are solely those of the authors and do not necessarily represent those of their affiliated organizations, or those of the publisher, the editors and the reviewers. Any product that may be evaluated in this article, or claim that may be made by its manufacturer, is not guaranteed or endorsed by the publisher.

## Footnotes

^1^ https://qiime2.org
