# Supplementary material for: Particles act as ‘specialty centers’ with expanded enzymatic function throughout the water column in the western North Atlantic
Source: Front Microbiol. 2022 Sep 28;13:882333. doi: 10.3389/fmicb.2022.882333 (PMC9553992; doi:10.3389/fmicb.2022.882333)
Supplement: Supplementary file 1 [file Data_Sheet_1.pdf]

## *Supplementary Material*

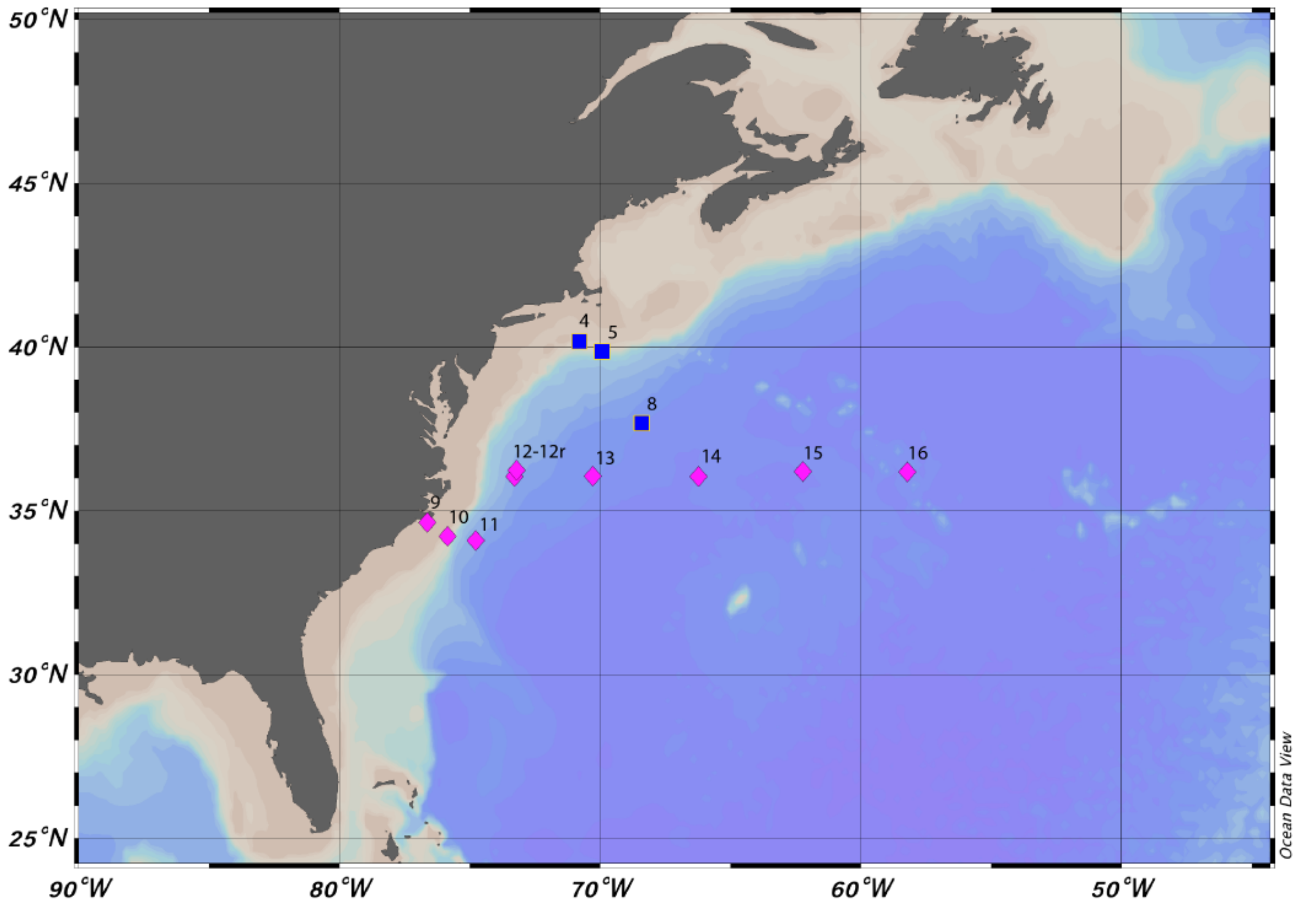


**Supplementary Figure 1.** Cruise stations aboard the *R/V Endeavor* during Spring 2015 (blue squares) and Spring 2016 (pink diamonds). Station locations are separated into coastal (Stns. 4, 9, and 10) and offshore (Stns. 5, 8, and 11–16).

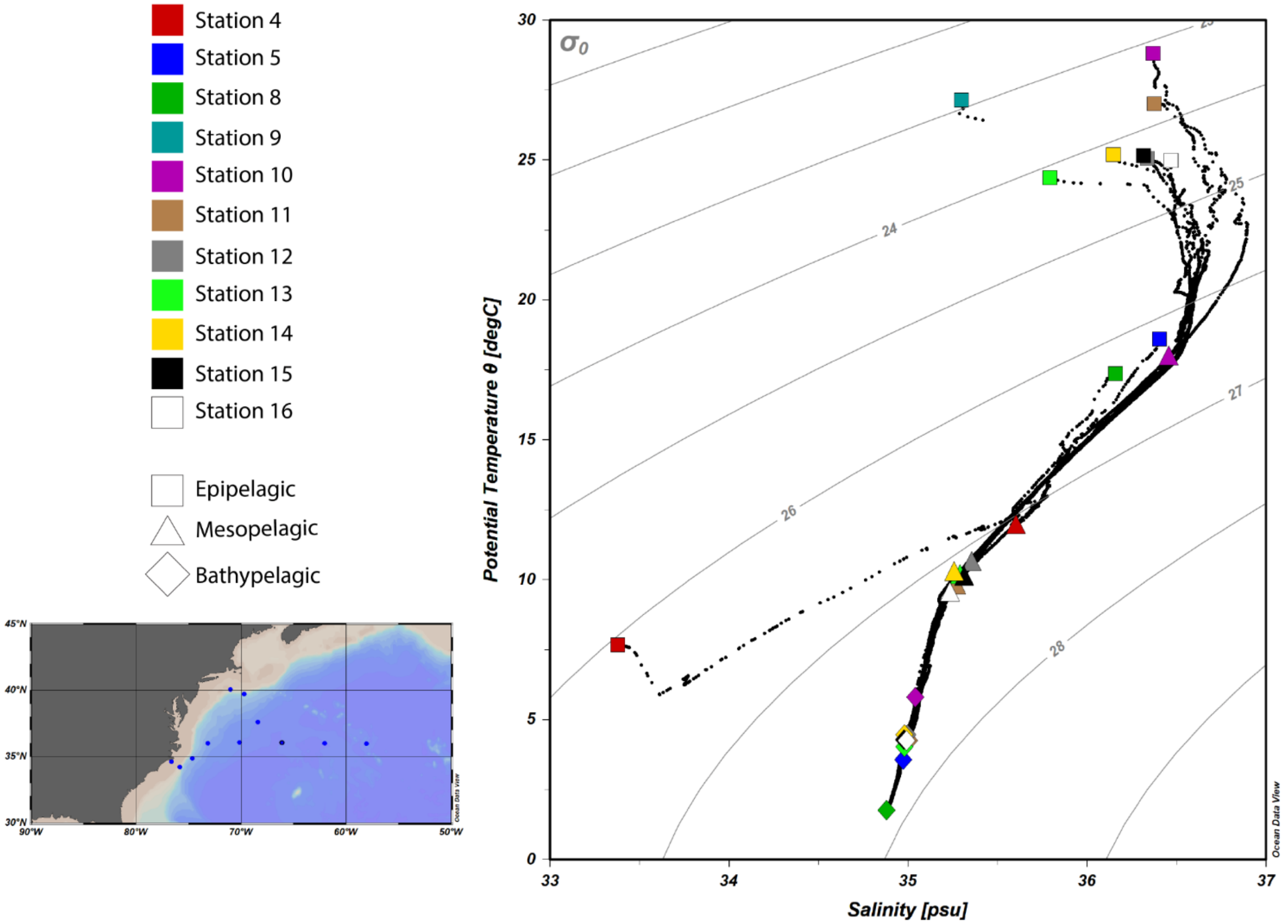


**Supplementary Figure 2.** Temperature/Salinity (T/S) diagram of water collected at the different stations. Stations are denoted by color and depths are differentiated by shape.

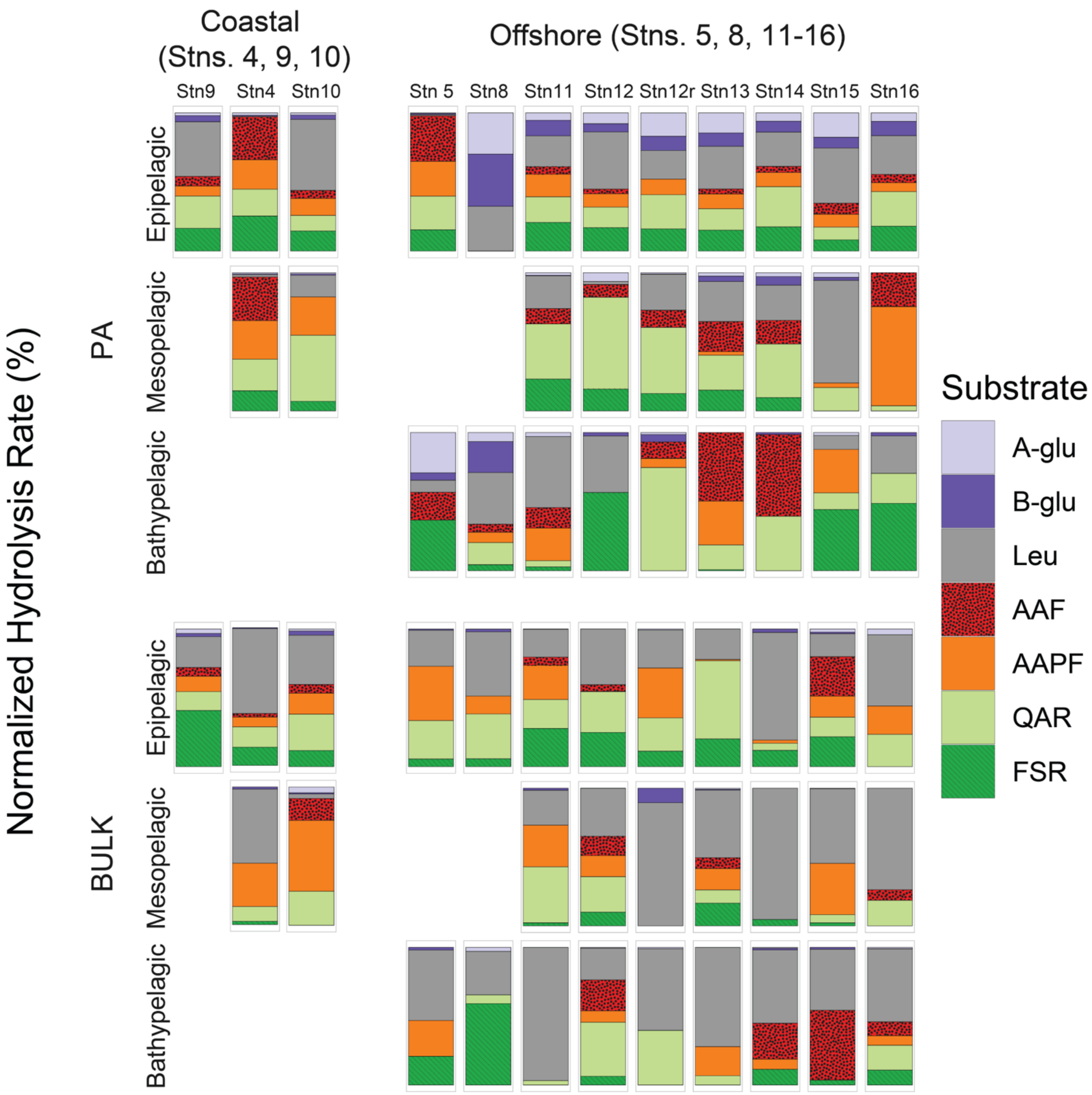


**Supplementary Figure 3.** Normalized hydrolysis rate for glucosidase and peptidase enzymatic activities. All activities are normalized to 100 percent for each station/depth. A-glu = alpha-glucosidase; B-glu = beta-glucosidase; Leu = leucine aminopeptidase; AAF = alanine-alanine-

phenylalanine-chymotrypsin; AAPF = alanine-alanine-proline-phenylalanine-chymotrypsin; QAR = glutamine-alanine-arginine-trypsin; FSR = phenylalanine-serine-arginine-trypsin.

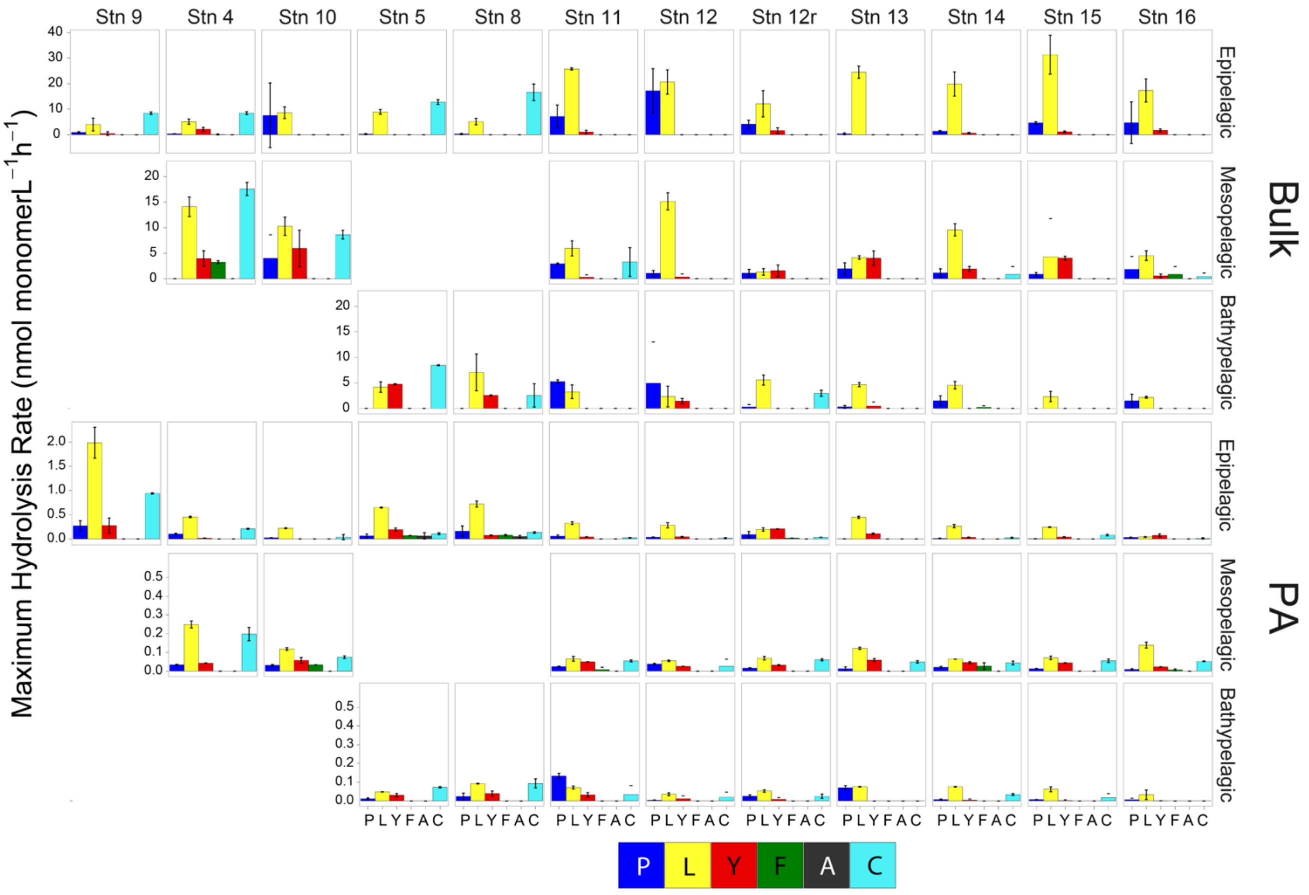


**Supplementary Figure 4.** Polysaccharide hydrolase activities for particle-associated (PA) and for unfiltered, bulk water samples at each station and depth. Note the difference in y-axis between each depth and between bulk and PA samples. P = pullulan; L = laminarin; X = xylan; F = fucoidan; A = arabinogalactan; C = chondroitin sulfate.

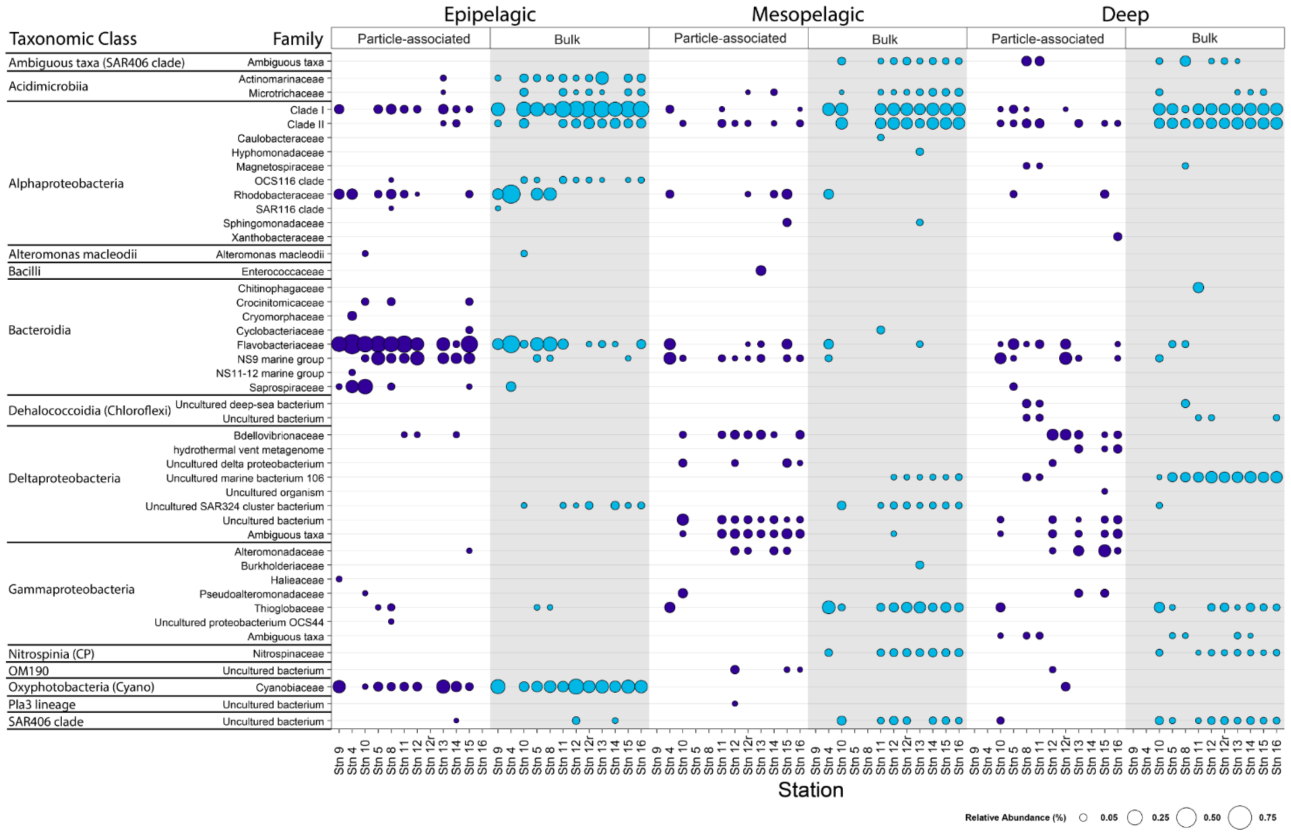


**Supplementary Figure 5.** Family-level composition of bulk (unfiltered) and PA (particle-associated) bacterial communities. Circle size correlates with relative abundance. Data shown was filtered and represents classes with ≥3% relative abundance in the data set.

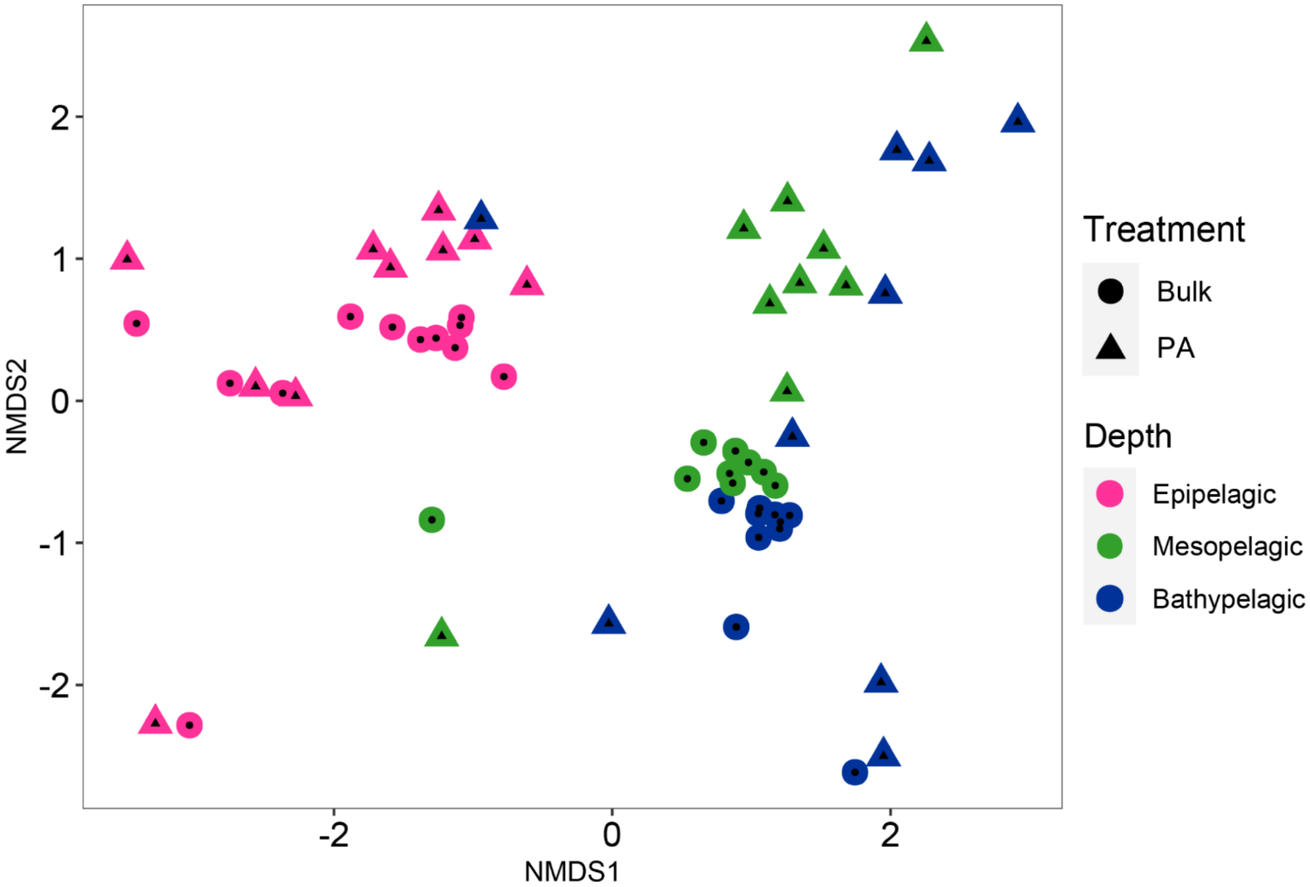


**Supplementary Figure 6.** Non-metric multidimensional scaling (NMDS) of the overall community composition based on the Bray-Curtis dissimilarity index. PA data are represented by triangles; bulk data are represented by circles. Surface depths are pink; mesopelagic depths are green; bathypelagic depths are blue.

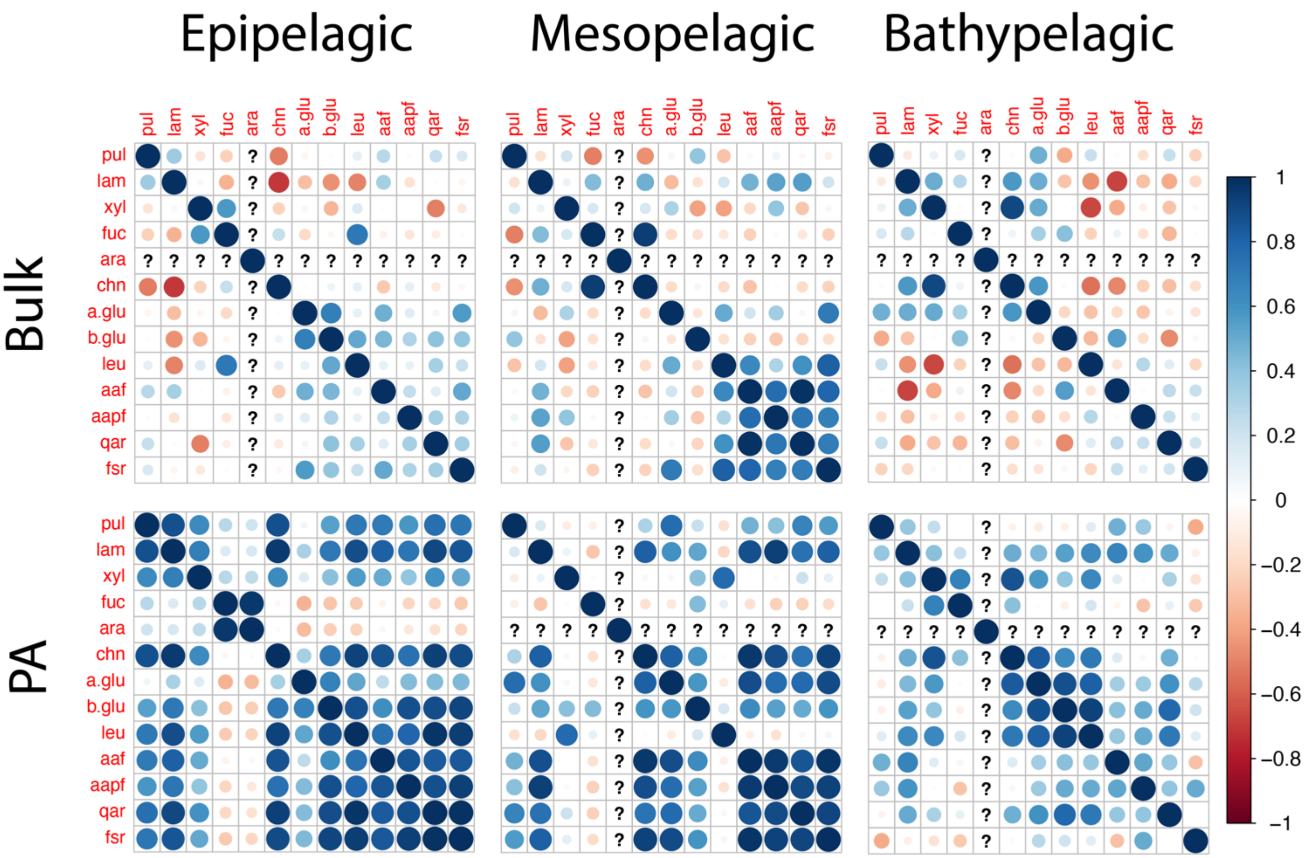


**Supplementary Figure 7.** Correlation plot displaying the Pearson correlation between enzymatic activity measurements for bulk and PA samples, separated by depth. Blue denotes positive correlation while red denotes negative correlations. The size of the circle as well as the shade of the color emphasizes the extent of correlations between different enzymatic activities. ? denotes that no activities were measured for that given substrate (i.e., arabinogalactan in many cases).

**Table S1**. Environmental data for collected water.

| Stn | Depth (m) | Salinity (psu) | Temperature (°C) | Oxygen (ml/L) | Chl-a (mg/m$^3$) |
|---|---|---|---|---|---|
| 4 | 2.2 | 33.3857 | 7.6468 | 7.296 | 0.8601 |
| | 199 | 35.5938 | 11.9835 | 4.6928 | 0.3459 |
| 5 | 2.1 | 36.4112 | 18.6171 | 5.0852 | 2.2231 |
| | 1977 | 34.9561 | 3.6719 | 5.7977 | 0.3893 |
| 8 | 5 | 36.1638 | 17.402 | 5.0827 | 0.9072 |
| | 4574 | 34.8784 | 2.1989 | 5.6156 | 0.2754 |
| 9 | 2 | 35.3048 | 27.1239 | 4.8648 | 0.8386 |
| 10 | 90 | 36.3611 | 28.8302 | 4.4845 | 0.1527 |
| | 300 | 36.4495 | 17.9508 | 3.4173 | 0.1276 |
| | 530 | 35.06 | 6.5279 | 4.4404 | 0.135 |
| 11 | 72 | 36.6829 | 22.6814 | 4.8057 | 0.5293 |
| | 842 | 35.2743 | 9.8646 | 3.3127 | 0.1219 |
| | 1500 | 35.0037 | 4.4031 | 5.7138 | 0.1224 |
| 12 | 95 | 36.6338 | 20.9448 | 5.0276 | 0.3592 |
| | 850 | 35.348 | 10.6375 | 3.2011 | 0.1404 |
| | 1500 | 35.0011 | 4.5326 | 5.6738 | 0.1625 |
| 12r | 131 | 36.5974 | 20.3848 | 4.8807 | 0.4828 |
| | 850 | 35.3004 | 10.2813 | 3.1107 | 0.1534 |
| | 1500 | 34.9985 | 4.5172 | 5.668 | 0.1518 |
| 13 | 95 | 36.5552 | 21.2651 | 4.9503 | 0.4823 |
| | 645 | 35.289 | 10.1261 | 3.128 | 0.1383 |
| | 1500 | 34.9802 | 4.1762 | 5.7922 | 0.1309 |
| 14 | 99 | 36.6399 | 20.9943 | 4.6907 | 0.1551 |
| | 900 | 35.2726 | 10.3001 | 2.9282 | 0.1377 |
| | 1500 | 34.9983 | 4.6176 | 5.6288 | 0.1266 |
| 15 | 82 | 36.6226 | 22.4731 | 4.6885 | 0.6715 |
| | 840 | 35.2915 | 10.1439 | 3.1939 | 0.1537 |
| | 1500 | 34.9848 | 4.4244 | 5.7504 | 0.1558 |
| 16 | 112 | 36.5632 | 20.0673 | 4.906 | 0.3634 |
| | 875 | 35.2301 | 9.5831 | 3.229 | 0.167 |
| | 1500 | 34.9956 | 4.5207 | 5.727 | 0.1691 |

**Table S2.** Incubation data for experimental setup.

| Stn | Depth (m) | Water filtered (L) FLA samples | Water filtered (L) -MCA samples | Water filtered (L) -MUF samples | In situ temperature (C) | GF setup incubation temperature (C) | Subsampling incubation temperature (C) |
|---|---|---|---|---|---|---|---|
| 4 | 2.2 | 4.7 | 4.5 | 7.8 | 7.7 | 4 | 8.5 |
| | 199 | 9.9 | 10.4 | 8.8 | 12 | 4 | 14 |
| 5 | 2.1 | 4.2 | 8.3 | 3.8 | 18.5 | 21 | 21 |
| | 1977 | 18 | 18.4 | 15.9 | 3.7 | 4 | 4 |
| 8 | 5 | 6.5 | 2.4 | 5.5 | 16.6 | 21 | 21 |
| | 4574 | 10.9 | 7.2 | 8.4 | 2.2 | 4 | 4 |
| 9 | 2 | 3 | 2.2 | 2.2 | 28.6 | 21 | 21 |
| 10 | 90 | 15.1 | 15.3 | 16 | 27 | 21 | 21 |
| | 300 | 17.7 | 19.2 | 19.4 | 18 | 21 | 16 |
| | 530 | 18.9 | 17.7 | 17 | 8 | 4 | 8 |
| 11 | 72 | 15.1 | 11.7 | 14 | 23 | 21 | 21 |
| | 842 | 17.7 | 17.6 | 18 | 10 | 21 | 12 |
| | 1500 | 11.7 | 13.8 | 15.2 | 4.9 | 4 | 4 |
| 12 | 95 | 17 | 16.9 | 15.7 | 20.6 | 21 | 21 |
| | 850 | 17.1 | 16.1 | 16.1 | 10 | 21 | 12 |
| | 1500 | 15.6 | 16.3 | 17.3 | 4.5 | 4 | 4 |
| 12r | 131 | 14.4 | 16.7 | 15.1 | 20.3 | 21 | 21 |
| | 850 | 18.4 | 19.1 | 17.9 | 10.1 | 21 | 12 |
| | 1500 | 15.4 | 17.9 | 17.8 | 4.5 | 4 | 4 |
| 13 | 95 | 12.4 | 13.2 | 13.5 | 21 | 21 | 21 |
| | 645 | 14.4 | 18.4 | 19 | 10.2 | 21 | 12 |
| | 1500 | 17.1 | 16.7 | 18.2 | 4.2 | 4 | 4 |
| 14 | 99 | 15.8 | 14.2 | 14.5 | 21.3 | 21 | 21 |
| | 900 | 17.7 | 19.5 | 19.7 | 10.1 | 21 | 12 |
| | 1500 | 17 | 18 | 18.6 | 4.7 | 4 | 4 |
| 15 | 82 | 14.6 | 14.8 | 13 | 23.2 | 21 | 21 |
| | 840 | 17.2 | 17.3 | 17.2 | 10.1 | 21 | 12 |
| | 1500 | 19.1 | 19.4 | 20 | 4.4 | 4 | 4 |
| 16 | 112 | 15.7 | 16.1 | 16.9 | 20.1 | 21 | 21 |
| | 875 | 18 | 16.7 | 17.2 | 9.8 | 21 | 12 |
| | 1500 | 18.9 | 19.1 | 18.4 | 4.5 | 4 | 4 |
